# Cortical suspensory button fixation has superior biomechanical properties to knotless anchor suture in anterior cruciate ligament repair: a biomechanical study

**DOI:** 10.1038/s41598-023-34766-9

**Published:** 2023-05-10

**Authors:** Thun Itthipanichpong, Napatpong Thamrongskulsiri, Pairat Tangpornprasert, Chanyaphan Virulsri, Danaithep Limskul, Somsak Kuptniratsaikul, Thanathep Tanpowpong

**Affiliations:** 1 Department of Orthopaedics, Faculty of Medicine, Chulalongkorn University and King Chulalongkorn Memorial Hospital, The Thai Red Cross Society, Rama IV Rd, Khwaeng Pathum Wan, Khet Pathum Wan, Krung Thep Maha Nakhon, Bangkok, 10330 Thailand; 2 Department of Anatomy, Faculty of Medicine, Chulalongkorn University and King Chulalongkorn Memorial Hospital, The Thai Red Cross Society, Bangkok, Thailand; 3 Center of Excellence for Prosthetic and Orthopedic Implant, Department of Mechanical Engineering, Faculty of Engineering, Chulalongkorn University, Bangkok, Thailand

**Keywords:** Medical research, Translational research, Orthopaedics

## Abstract

The purpose of our biomechanical study was to assess load-to-failure, stiffness, gap formation following cyclic loading, and the failure mechanism for anterior cruciate ligament (ACL) repair comparing the cortical suspensory button and knotless anchor suture. Eight Thiel’s embalmed paired cadaveric knees from four cadavers were dissected. The specimens were assigned to undergo ACL repair either with cortical suspensory button or with knotless anchor suture. The Instron machine replicates cyclic loading and then determines the gap formation. Traction was applied until failure. The load-to-failure, stiffness, and modes of failure in both groups were recorded. The load-to-failure, stiffness, and gap formation were compared between the two groups using the student's t-test. The mean load-to-failure in the cortical suspensory button group was significantly higher than the knotless anchor suture group (212.96 ± 54.57 vs 44.57 ± 20.80, *p* value < 0.01). No statistically significant difference was found regarding gap formation following cyclic loading and stiffness between the cortical suspensory button group and the knotless anchor suture group. This biomechanical study showed a higher load-to-failure for the ACL repair with cortical suspensory button compared to ACL repair with knotless anchor suture, while no statistically significant difference was found regarding the gap formation following cyclic loading and the stiffness. The load-to-failure in both cortical suspensory button and knotless anchor suture are below regular daily activity load. Thus, an internal brace or external support is recommended during rehabilitation.

## Introduction

Anterior cruciate ligament (ACL) tears are the most common knee injury associated with sports. Annually, between 100,000 and 200,000 ACL injuries occur in the United States, with football, skiing, and gymnastics being the most prevalent sources of injuries^1^. Historically, ACL injuries were treated with primary ACL repair. More recent research has shown that ACL reconstruction is more successful than primary ACL repair. ACL reconstruction is now the gold standard for ACL injury treatment^2–5^.

In 1895, Mayo Robson^4^ reported the first recorded case of anterior cruciate ligament (ACL) repair in a 41-year-old male who underwent open primary repair of bilateral ACL tears at the femoral attachment site. In 1976, Feagin and Curl^3^ conducted a study on athletes who underwent open primary ACL repair, and found that most patients were able to return to sports. However, after a five-year follow-up, the failure rate was high with 94% of patients experiencing instability, 53% experiencing reinjury, and 34% requiring a second surgical treatment. Sherman et al.^6^ introduced a four-level classification system in 1991. Type 1 involves a complete tear of the anterior cruciate ligament (ACL) from the femoral attachment, with no remaining connection to the femur. Type 2 includes injuries where less than 20% of ligaments remain attached to the femoral attachment. Type 3 tears occur when less than 33% of ligaments are connected to the femoral attachment. Type 4 refers to a mid-substance tear. With a reported follow-up of 61 months following open primary ACL repair, patients over the age of 22 with a ski injury, a Type 1 tear, good tissue quality, and a low-grade pivot had a favorable result.

Compared to primary ACL repair, there are several disadvantages of ACL reconstruction including the loss of the native knee kinematics, loss of proprioceptive sensation, inability to prevent osteoarthritis, and an increasing difficulty in subsequent surgeries^7^. Arthroscopic surgery has increased in popularity over the past decade with advancements in surgical equipment. Arthroscopic ACL primary repair has received increased attention and has been reported to have favorable surgical outcomes in the short- and medium-term, particularly in patients with Sherman Type 1 ACL tear^8,9^.

Each orthopedic surgeon employs a unique approach and suture equipment when doing arthroscopic primary ACL repair^7,10–16^. Cortical suspensory button and knotless anchor suture are the most frequently used implants. The cortical suspensory button has been employed by certain surgeons to reestablish the connection between a ruptured ligament and the femoral footprint^12,15,16^. Heusdens et al. have recently reported on the results of a two-year follow-up investigation of anterior cruciate ligament (ACL) reconstruction utilizing the cortical suspensory button, and have identified substantial enhancement in clinical outcomes^17^. Meanwhile, some surgeons also utilize the knotless suture anchor^11,13,14^, and have found promising results in their procedures^18,19^.

However, there are no biomechanical comparisons between an ACL repair with a cortical suspensory button and a knotless anchor suture to evaluate whether they are stronger and robust enough to resist the forces applied on the knee during postoperative rehabilitation. To date, there has been no biomechanical study completed on human cadaveric knees. There is only one study using fresh frozen porcine knees^20^. The objective of this study was to determine the load-to-failure (N), stiffness (N/mm), gap formation (mm), and failure mechanism for ACL repair with both cortical suspensory button and knotless anchor suture repair techniques.

## Materials and methods

### Sample size calculation

Calculations of sample size are based on results from research performed on porcine ligaments^20^. Epitools (Ausvet, Australia) was used to calculate a total sample size of 6 and a sample size of 3 per group with confidence level of 0.95, and power = 0.8. To avoid missing or incomplete data, a 33% increase in sample size was added to the total sample size of 8 or the sample size of 4 per group.

### Inclusions

The study was approved by the Chulalongkorn University Faculty of Medicine's Institutional Review Board (IRB No. 632/64). Informed consent was obtained from all subjects and/or their legal guardians for using cadaveric samples used in the study. Eight paired cadaveric knees were taken from four Thiel’s embalmed cadavers^21–23^. Specimens with altered knee anatomy from any pathology were excluded. Cadaver demographic data, such as age, weight, height, and gender, has been obtained from the Chula soft cadaver surgical training center's registry. The present study was conducted in accordance with the tenets of the Declaration of Helsinki, 1975, as revised in 2013.

### Study procedures

**Figure 1:**
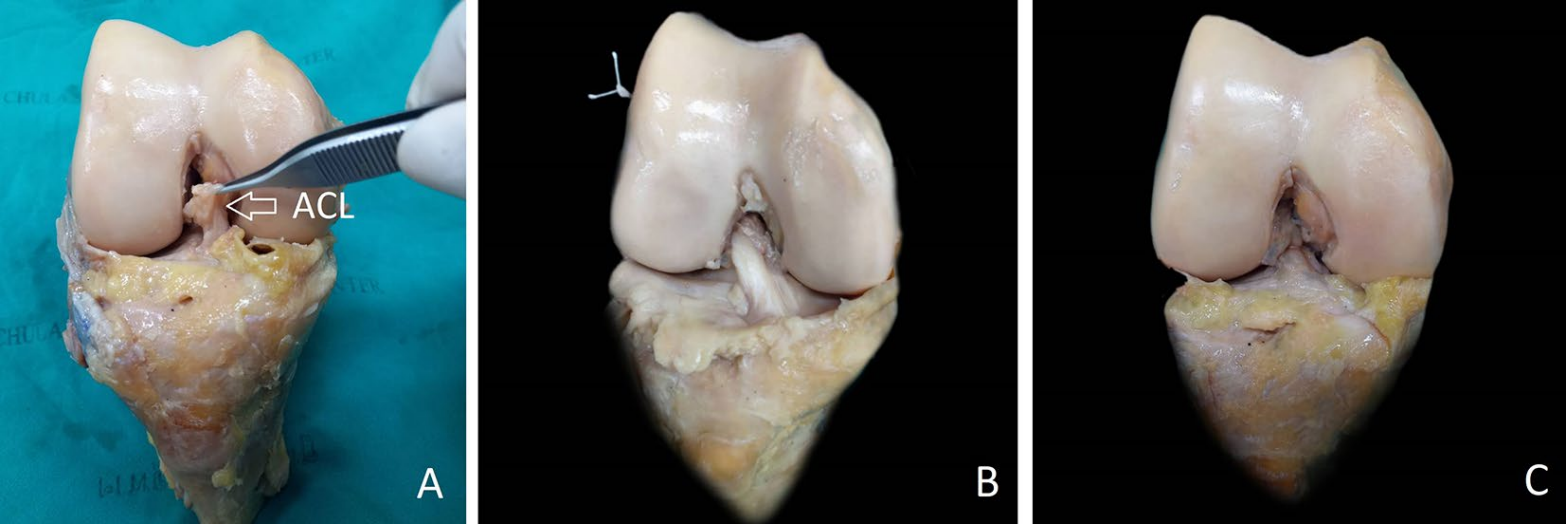
(**A**) An ACL was peeled off the femoral attachment to simulate Sherman type 1 tear. (**B**) ACL was repaired with cortical suspensory button technique, (**C**) ACL was repaired with knotless anchor suture technique. (*ACL* anterior cruciate ligament).

Eight Thiel’s embalmed paired cadaveric knees were prepared^21–23^. The femur and tibia were cut fifteen centimeters from the joint line. The collateral and posterior cruciate ligaments were peeled off, leaving only the anterior cruciate ligament connected between femur and tibia. A type 1 ACL tear was created in each cadaveric knee. An ACL is peeled off the femoral attachment (Fig. 1A) and is repaired using a cortical suspensory button (CSB) or knotless anchor suture (KAS).

### Repair with cortical suspensory button technique (CSB)

The ACL was sutured using #2 HiFi suture (CONMED, Utica, NY) with a single loop stitch. After stitching the ACL, a 4.5-mm guide-reamer was used to create the ACL femoral footprint. All sutures were threaded through the femoral footprint holes. The cortical suspensory button (XO button, CONMED, Utica, NY) was placed at the lateral femoral cortex with a surgical knot and five half-hitches with the ACL remnant tension in the semi-extension position (Fig. 1B).

### Repair with knotless anchor suture technique (KAS)

To repair the ACL using the knotless anchor suture technique, stitch ACL was performed using the cortical suspensory button technique was completed and followed by drill and tap of 4.5 mm × 20 mm holes in the femoral footprint of the ACL. Threading of all the sutured limbs through the eyelet of the knotless anchor suture (4.5-mm PopLok, CONMED, Utica, NY) and insertion of the knotless anchor suture into the prepared ACL femoral footprint with the ACL remnant tension in the semi-extension position was performed. The end of the sutures were then cut-off (Fig. 1C).

### Model for testing

**Figure 2:**
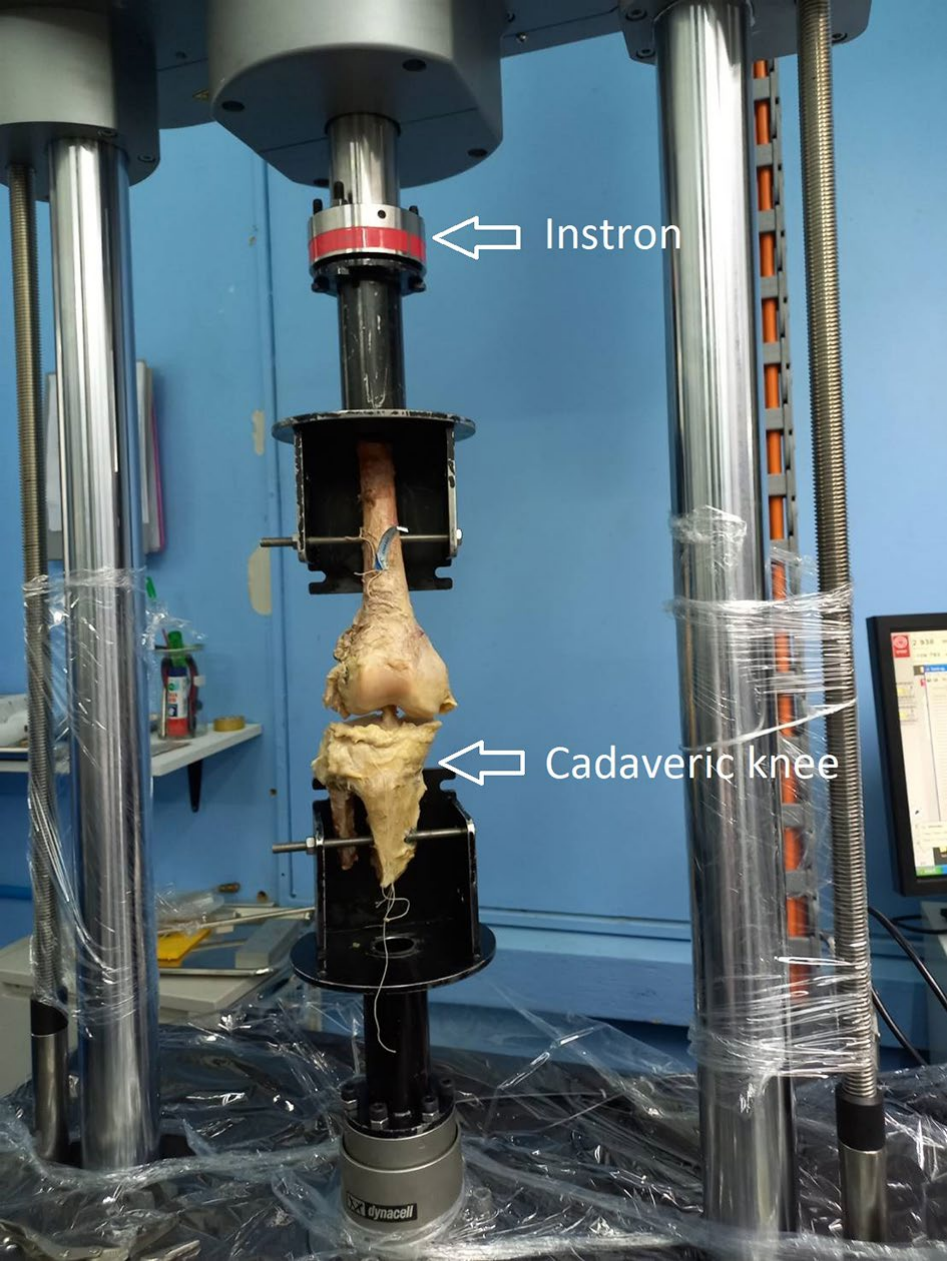
Experimental setup with testing machine (E10000, Instron, Canton, MA).

Biomechanical testing was performed on specimens using a mechanical testing machine (E10000, Instron, Canton, MA) with the tibia attached to the base stationary portion and the femur attached to a servohydrolic testing system in a 180-degree knee-upright position (semi-extension) (Fig. 2). While exerting force on the femur, the Tibia bone remained at rest. The Servohydrolic testing system (E10000, Instron, Canton, MA) replicates cyclic loading in a position-controlled mode. The testing started with 500 cycles at 0.75 Hz and a peak elongation of 1 mm. Determination of the gap formation (plastic deformity) after 500 cycles was completed by increasing peak elongation from 1 to 3 mm. Peak elongation was then increased to 5 mm (1500 cyclic loading cycles) and the gap formation was monitored every 500 cycles throughout the cyclic loading. Traction was applied at a rate of 50 mm/min until failure. The load-to-failure and stiffness between the pull-to-failure and primary modes of failure in both groups were measured.

### Outcome measurement

Biomechanical evaluation of the ACL repair techniques using a cortical suspensory button and a knotless anchor suture. Key outcomes measured were load-to-failure (N), stiffness (N/mm), gap formation (mm).

### Statistical analysis

Statistical analysis was performed using SPSS 22.0 (IBM, USA) for Windows. The student's t-test was used to compare the load-to-failure (N), stiffness (N/mm), and gap formation (mm) values between the two groups. The 95% confidence interval was also calculated for both groups. The significance level was set at *p* value ≤ 0.05.

## Results

Eight of the specimens, one knee from each cadaver in each group, came from four cadavers. The cadavers had an average age of 68.00 ± 17.40 years, average body weight of 65.00 ± 10.80 kg, and average height of 171.25 ± 12.50 cm. The specimens were 3 males and 1 female. The details of the demographic data are shown in Table 1.

**Table 1:** Demographic data (kg: kilogram; cm: centimeter; *SD* standard deviation).

Cadaver number	Age (years)	Body weight (kg)	Height (cm)	Gender
1	77	65	170	Male
2	85	75	185	Male
3	45	70	175	Male
4	65	50	155	Female
Mean ± SD	68.00 ± 17.40	65.00 ± 10.80	171.25 ± 12.50	

At 1-, 3-, and 5-mm peak elongation, the mean gap formation in the CSB group was 0.96 ± 0.18 mm, 2.14 ± 0.71 mm, and 3.92 ± 0.41 mm, respectively. In comparison, the mean gap formation of KAS group at 1, 3, and 5 mm was 1.03 ± 1.09 mm, 2.51 ± 0.75 mm, and 4.41 ± 0.71 mm, respectively. There is no significant difference between the CSB and KAS groups in the cyclic data at the end of each cycle up to 5 mm (Table 2). During cycle loading, none of the specimens failed. As a result, all of the specimens were put through a final pull-to-failure test.

**Table 2:** Gap formation (mm: millimeter; CSB: cortical suspensory button; KAS: knotless anchor suture; 95% CI: 95% confidence interval).

Peak elongation (mm)	Gap formation		
	1 mm	3 mm	5 mm
CSB	0.96 ± 0.18	2.14 ± 0.71	3.92 ± 0.41
KAS	1.03 ± 1.09	2.51 ± 0.75	4.41 ± 0.71
	*p* value = 0.9100 95% CI − 1.2845 to 1.4145	*p* value = 0.4952 95% CI − 0.8891 to 1.6391	*p* value = 0.2797 95% CI − 0.5138 to 1.4838

The CSB group had a mean load-to-failure and stiffness of 212.96 ± 54.57 N and 34.83 ± 9.40 N/mm, respectively. The mean load-to-failure and stiffness of the KAS group was 44.57 ± 20.80 N and 28.76 ± 14.48 N/mm. There was a significant difference in load-to-failure between the CSB and KAS groups (*p* value < 0.01) (Table 3).

**Table 3:** Load-to-failure and stiffness (N: Newton; mm: millimeter; CSB: cortical suspensory button; KAS: knotless anchor suture; 95% CI: 95% confidence interval; *: statistically significant).

	Load-to-failure (N)	Stiffness (N/mm)
CSB	212.96 ± 54.57	34.83 ± 9.40
KAS	44.57 ± 20.80	28.76 ± 14.48
	*p* value = 0.0012* 95% CI: − 239.8384 to − 96.9466	*p* value = 0.5077 95% CI − 27.1941 to 15.0391

The entire CSB group failed due to knot slippage at the button. Three specimens of the KAS group failed due to suture slippage from the anchor. Another failed due to a mid-substance ACL tear (Table 4).

**Table 4:** Failure mechanism (*CSB* cortical suspensory button, *KAS* knotless anchor suture, *ACL* anterior cruciate ligament).

Specimens number	Failure mechanism
1 (CSB)	Knot slippage at the button
2 (KAS)	Suture slippage from the anchor
3 (CSB)	Knot slippage at the button
4 (KAS)	Suture slippage from the anchor
5 (CSB)	Knot slippage at the button
6 (KAS)	ACL mid-substance tear
7 (CSB)	Knot slippage at the button
8 (KAS)	Suture slippage from the anchor

## Discussion

A published meta-analysis found minimal complications and high functional scores following ACL repair, but it is limited by short follow-up and a significant risk of selection and publication bias^24^. Some studies have also found that adolescents have a higher risk of re-rupture due to high activity and an early return to sports^25,26^. We performed the study to determine load-to-failure, the stiffness, gap formation, and failure mechanism for ACL repair with cortical suspensory button and knotless anchor suture.

With values of 212.96 ± 54.57 N, the CSB group had the highest load-to-failure. The stiffness of the CSB group was 34.83 ± 9.40 N/mm. The KAS group’s mean load-to-failure and stiffness were 44.57 ± 20.80 N and 28.76 ± 14.48 N/mm, respectively. Between the CSB and KAS groups, there was a significant difference in the mean load-to-failure (*p* value < 0.01). Bachmaier et al. published a biomechanical study on fresh frozen porcine knees showing that adjustable single-cinch cortex button fixation had the highest ultimate strength when compared to knotless anchor suture, double cinch-fixed loop cortical button, and single cinch-fixed loop cortical button fixation^20^. According to our findings in human cadaveric knees, most of construct failures were caused by knot slippage at the button and suture slippage from the anchor. It is possible to sew the ACL with 1 or 2 loops because failure is not related to the repair site. The present biomechanical found no significant difference in gap formation after cyclic loading between CSB and KAS groups. Although the cadavers used in our study were of advanced age and concerns regarding bone quality were present, our experimental results revealed that none of the specimens failed due to bone breakage or anchor pull-out. This implies that the bone quality was still adequate.

Morrison analyzed ACL loads during activities of daily living and found that normal level walking created 169 N of force, while descending stairs generated 445 N of force due to the activation of the knee extensor mechanism. Ascending stairs, on the other hand, produced forces of less than 100 N^27–29^. Based on the findings of the present study, the load-to-failure values for the cortical suspensory button and knotless anchor suture femoral fixation were found to be 212.96 ± 54.57 N and 44.57 ± 20.80 N, respectively. According to a biomechanical study of ACL repair with and without internal brace augmentation by Massey et al., ACL repair with internal brace augmentation had a load-to-failure of 693 ± 248 N and a load-to-failure of 279 ± 91 N without augmentation^30^. Kuptniratsaikul et al.^12^, reported a surgical technique to augment the ACL with multiple high strength sutures which is comparable to internal bracing with suture tape. As a result, if the ACL is repaired with femoral fixation using a cortical suspensory button or knotless anchor suture, reinforcement with synthetic sutures or protection with an internal brace is recommended.

Heusdens et al.^17^ reported the 2-year follow-up results of a novel technique for repairing acute, proximal ACL tears using a cortical suspensory button and suture tape augmentation. The study included 42 patients with good ACL tissue quality and excluded those with poor tissue quality, retracted ACL remnants, or multiple ligament injuries. The results showed significant improvements in the Knee Injury and Osteoarthritis Outcome Score (KOOS), the Visual Analogue Pain Scale, and the Veterans RAND 12-Item Health Survey physical score, with meaningful changes in the KOOS sport and recreation subscale. However, the Marx activity scale decreased significantly, and two patients (4.8%) reported ACL rupture. Jonkergouw et al.^18^ examined the outcomes of arthroscopic primary repair using knotless suture anchor of proximal ACL tears in a cohort of 56 patients, followed up for a minimum of 3.2 years. Comparing internal brace (27 patients) and without internal brace (29 patients), they found that arthroscopic primary repair using knotless suture anchor with or without internal brace resulted in good objective and subjective outcomes and similar outcomes. Vermeijden et al.^19^ reported the study of the same cohort, which aimed to compare the extent to which patients forget about their operative knee joint following arthroscopic primary repair versus reconstruction of the ACL. Patients who underwent primary repair reported less daily awareness of their operated knee compared to those who underwent reconstruction. These findings were more significant in patients who were older than 30 years, male, and had a body mass index greater than 25. It is currently believed that in the short-term, the outcomes of ACL repair are similar to those of repair or reconstruction, regardless of the type of repair technique used. However, there is limited evidence regarding long-term outcomes and complications.

All of the examples in CSB failed because the knot slipped on the femoral site. This means that the construction of the knot is essential if the cortical button is chosen for ACL repair. On the other hand, KAS placed more reliance on the stability of the attachment between the suture and the anchor. However, neither method is strong enough for everyday tasks. Therefore, it is advised to wear external support for the first few weeks following the operation; alternatively, an internal brace can be performed in conjunction with ACL repair.

### Limitations

This study has several limitations. First, the loads were pulled vertically along the longitudinal axis, resembling the worst-case scenario rather than anterior translation or pivot-shifting. Second, Thiel's embalmed cadavers were used in this study, rather than fresh frozen cadavers, which have the same elasticity, color, and flexibility as in vivo ligaments. Studies have shown that the Thiel embalming method is effective for preserving ligaments for research purposes^21,23^. Third, our cadavers' average had wide range, with an average age of 68.00 ± 17.40 years which does not properly represent the younger population for whom ACL repair operations are commonly performed, and the quality of the bones and ligaments may deteriorate as a result of age. The results of our study suggest that despite concerns about the bone quality of the cadavers used, the absence of failures due to bone breakage or anchor pull-out implies that the bone quality was still adequate. This finding has important implications for the use of cadaveric specimens in biomechanical studies, particularly in cases where concerns about bone quality may limit their use. However, further research is needed to confirm these findings and explore the potential impact of bone quality on biomechanical outcomes in other contexts. Fourth, this study had a small sample size which limited finding significant differences. Finally, only one manufacturer (CONMED, Utica, NY) of knotless anchor suture and cortical suspensory button was tested in this study.

## Conclusions

This study showed a higher load-to-failure for the ACL repair with cortical suspensory button compared to ACL repair with knotless anchor sutures. However, the load-to-failure in both cortical suspensory button and knotless anchor suture are below a regular daily activity load. An internal brace or external support is recommended during rehabilitation.

## Data Availability

The datasets used and analyzed during the current study are available from the corresponding author on reasonable request.
